# Improving the Spatial Prediction of Soil Organic Carbon Stocks in a Complex Tropical Mountain Landscape by Methodological Specifications in Machine Learning Approaches

**DOI:** 10.1371/journal.pone.0153673

**Published:** 2016-04-29

**Authors:** Mareike Ließ, Johannes Schmidt, Bruno Glaser

**Affiliations:** 1 Department of Soil Physics, Helmholtz Centre for Environmental Research–UFZ, Halle (Saale), Germany; 2 Department of Geosciences/ Soil Physics Division, University of Bayreuth, Bayreuth, Germany; 3 Department of Physics and Geosciences/ Institute of Geography, University of Leipzig, Leipzig, Germany; 4 Department of Soil Biochemistry, Martin-Luther-Universität Halle-Wittenberg, Halle (Saale), Germany; Qom University, ISLAMIC REPUBLIC OF IRAN

## Abstract

Tropical forests are significant carbon sinks and their soils’ carbon storage potential is immense. However, little is known about the soil organic carbon (SOC) stocks of tropical mountain areas whose complex soil-landscape and difficult accessibility pose a challenge to spatial analysis. The choice of methodology for spatial prediction is of high importance to improve the expected poor model results in case of low predictor-response correlations. Four aspects were considered to improve model performance in predicting SOC stocks of the organic layer of a tropical mountain forest landscape: Different spatial predictor settings, predictor selection strategies, various machine learning algorithms and model tuning. Five machine learning algorithms: random forests, artificial neural networks, multivariate adaptive regression splines, boosted regression trees and support vector machines were trained and tuned to predict SOC stocks from predictors derived from a digital elevation model and satellite image. Topographical predictors were calculated with a GIS search radius of 45 to 615 m. Finally, three predictor selection strategies were applied to the total set of 236 predictors. All machine learning algorithms—including the model tuning and predictor selection—were compared via five repetitions of a tenfold cross-validation. The boosted regression tree algorithm resulted in the overall best model. SOC stocks ranged between 0.2 to 17.7 kg m^-2^, displaying a huge variability with diffuse insolation and curvatures of different scale guiding the spatial pattern. Predictor selection and model tuning improved the models’ predictive performance in all five machine learning algorithms. The rather low number of selected predictors favours forward compared to backward selection procedures. Choosing predictors due to their indiviual performance was vanquished by the two procedures which accounted for predictor interaction.

## 1 Introduction

Tropical forests play a key role in the global carbon cycle storing a total of 471 Pg carbon [1,2]. The soils’ carbon storage potential is generally even greater than that of the vegetation [3]. Don et al. [4] report, that 36 to 60% of the tropical ecosystem’s carbon is stored in soil. But, land use change from primary forest to other land uses leads to a decrease in soil organic carbon (SOC) stocks [4,5]. Ecuador in particular has the highest annual deforestation rate in South America [6]. Tapia-Armijos et al. [7] report a reduction of the area covered by natural vegetation by 46% (Southern Ecuadorian provinces). Local farmers make “extensive use of fire” to convert primary forest into farming land and pastures [8]. According to Bahr et al. [9] 9 to 13 Mg SOC per hectare are lost due to land use changes from forest to crop land and pastures. Finally, spatial estimates of SOC are increasingly important to acknowledge the soils’ carbon storage potential in the context of climate change. However, it is particluarly the tropical mountain areas with their thick organic layers which are highly complex and difficult to access [10]. SOC stock data of tropical mountain forest soils are scarce, SOC stock data of the organic layer hardly exist.

Regression based digital soil mapping (DSM) provides a means of regionalising soil data from a limited amount of samples to a landscape level by making use of the factors of soil formation [11] as predictors. Spatial continuous predictors representing topography and vegetation are obtained from digital elevation models (DEMs) and satellite images. However, for many soil properties, spatial regression modelling may not produce a robust model. According to Ryan et al. [12], low r^2^ values may result from one or more of the following causes: (1) poor relation to the available environmental predictor variables, (2) extreme local variation due to unknown or random effects, or (3) the collected data spans a very small interval in the total range of the response variable. While the latter can be mostly avoided by a good sampling design which follows a good representation of the predictor space [13], the former two causes provide real challenges.

DEMs are often used at their original raster resolution with a 3x3 window size for the calculation of the derived predictors. However, a number of studies suggest that predictor-response relationships are strongly landscape and scale dependent [12]. Cavazzi et al.[14] investigated the interacting effect between window and raster cell size and found cell size to be significant in all considered areas whereas the interaction between window and cell size was significant in morphological rough areas. Finally, soil-forming factors (predictors) vary and respond at different scales [15]. Maynard and Johnson [16] found a strong scale-dependency for total carbon having the best model performance at coarse neighbourhood extents (150 to 300 m); DEM resolution affected soil-terrain correlations to a much lesser degree. Samuel-Rosa et al. [17] have shown that investigating the impact of scale in predictors is more important when the predictor-response relationships are weak. Finally, multi-scale as well as feature selection approaches according to [15] deserve more research to obtain a better prediction accuracy.

The term feature selection refers to the process of removing irrelevant predictors from the predictor set to enhance a model’s performance and generalisation capability (e.g. [18]). It requires an exhaustive search of all possible subsets of predictors in order to decide which subset performs best. With a large set of predictors, this procedure is simply not applicable [19]. Therefore, due to practicability reasons the selection of a subset which is just good enough if not optimal, might have to be sufficient [20]. Predictor selection procedures can be described by two main categories [21]: (1) filter methods and (2) wrapper methods. Filter methods make a predictor assessment based on general characteristics of the dataset (e.g. predictor–response correlation), independently from the particular machine learning algorithm and hence ignore the predictors’s effectiveness within the particular model. Wrapper methods, evaluate predictor performance by running the particular machine learning algorithm on the dataset [22]. Accordingly, most filter methods evaluate each predictor individually and ignore possibly important predictor interactions, whereas wrapper methods result in an increase in computation time [23]. It is often argued that particularly recursive partitioning methods do not require predictor selection as they are at least theoretically resistant to irrelevant predictors [23,24]. However, Witten and Frank [18] show that decision trees are affected by non-informative predictors and Kuhn and Johnson [23] show that ANN and SVM are affected to an even larger extent.

A number of machine learning algorithms are commonly used in DSM, such as e.g. tree-based methods, artificial neural networks (ANN), multivariate adaptive regression splines (MARS) and support vector machines (SVM). Each of them has its strengths and pitfalls and in dependence of the particular application and soil-landscape there is no single algorithm which serves all. Grimm et al. [25] and Guo et al. [26] use random forest to map SOC. Stepwise regression was applied by Gessler et al. [27], Gasparini et al. [28] and Zhang et al. [29]. Martin et al. [30,31] applied boosted regression trees. Pastick et al. [32] and Bou Kheir et al. [33] used decision tree models. ANN were applied by Dai et al. [34]. However, comparisons of various machine learning approaches to spatially predict SOC in tropical mountain areas are scarce. Indeed we only found one: Were et al. [35] compare SVM to ANN and random forest to map SOC stocks in an afromontane landscape with SVM showing the best performance.

Typically the machine learning algorithms have one or several tuning parameters, and the estimation of these tuning parameters should be based on an estimate of the prediction error [24]. The number of layers and neurons is a crucial decision to be made when constructing ANNs. A network with too few neurons cannot differentiate between complex data patterns, whereas too many neurons would lead to overfitting [36]. In support vector regression the model parameters C and ε as well as the kernel parameters must be tuned to obtain sensible results [37,38]. According to Hastie et al. [24] random forests do remarkably well with little tuning. In our experience [10,39] tuning random forest and boosted regression tree models can improve prediction results, but tuning needs to be tested carefully for it can also cause overfitting.

Particularly in the complex soil-landscape settings of tropical mountain areas, the choice of methodology for spatial prediction is of high importance to improve the expected poor model results. Accordingly within this study four aspects will be considered to obtain the best possible model:

1. Considering different spatial settings/scales for the predictors
2. Apply predictor selection strategies to reduce noise and enhance the model’s performance
3. Try different machine learning algorithms to capture the complex predictor-response relation. Among the vast choice of algorithms, we selected algorithms that follow different adaptation strategies
4. Apply model tuning to optimise the model’s performance

## 2 Material and Methods

### 2.1 Research area

**Fig 1:**
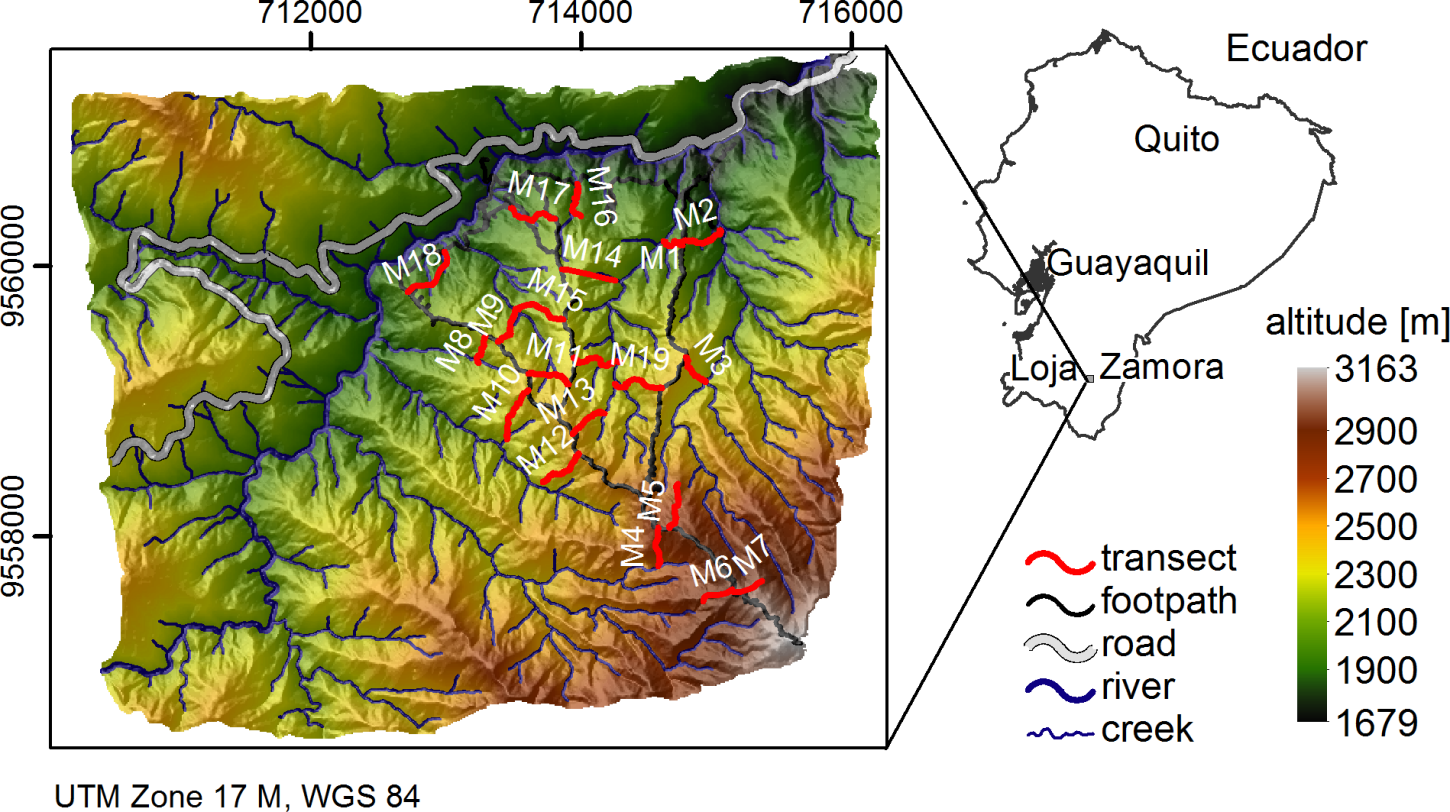
Research area with sampling transects (adapted from [45]).

The research permit for the respective area was granted by the Ecuadorian Ministry of the Environment. The research area is situated on the eastern escarpment of the southern Ecuadorean Andes between the provincial capitals Loja and Zamora (Fig 1). It forms part of the Natural Reserve Podocarpus—El Condor and is mainly covered by tropical montane forest vegetation changing into shrub and grassland vegetation above the tree line of ca. 2800 m a.s.l.. The area is influenced by an altitudinal decrease in temperature and increase in rainfall. This corresponds to a mean annual air temperature ranging from 19.4°C to 9.4°C and a mean annual total rainfall from 2050 mm to 4400 mm [40,41]. Geologically the area is part of a metamorphic belt of palaeozoic age. Litherland et al. [42] describe the underlying bedrock as part of the Chiguinda Unit of the litho-tectonic section Loja Terrane, consisting of pelite, schist, phyllite, meta-siltstone, sandstone and quartzite. The area is known for its immense organic layers leading to its description as sloping mire soil-landscape [10]. Its formation is partly related to the dominance of soil hydromorphic properties in the topsoil caused by the silty soil texture, heavy rainfall and shallow slope parallel subsurface flow [43,44]. The occurrence of frequent landslides adds to the complexity of this remote tropical mountain landscape.

### 2.2 Dataset

#### 2.2.1 Carbon data–sampling and analysis

The organic layer of the research area was sampled along transects as indicated in Fig 1. The transects were laid to cover the complete hillslope from the ridge to the valley bottom. They were positioned to cover terrain units formed by an overlay of two altitudinal, three slope and two aspect classes, while inclination had to be considered to permit accessibility of the very steep terrain [45]. Each transect was then sampled randomly at three positions to cover the upper, middle and foot slope. Sampling was conducted by using a 20 by 20 cm metal frame of 10 cm height. Samples were oven-dried at 45°C until mass consistency. After the removal of fresh roots, organic carbon contents were determined by a VARIO MAX elemental analyser at the soil laboratory of the University of Halle.

**Fig 2:**
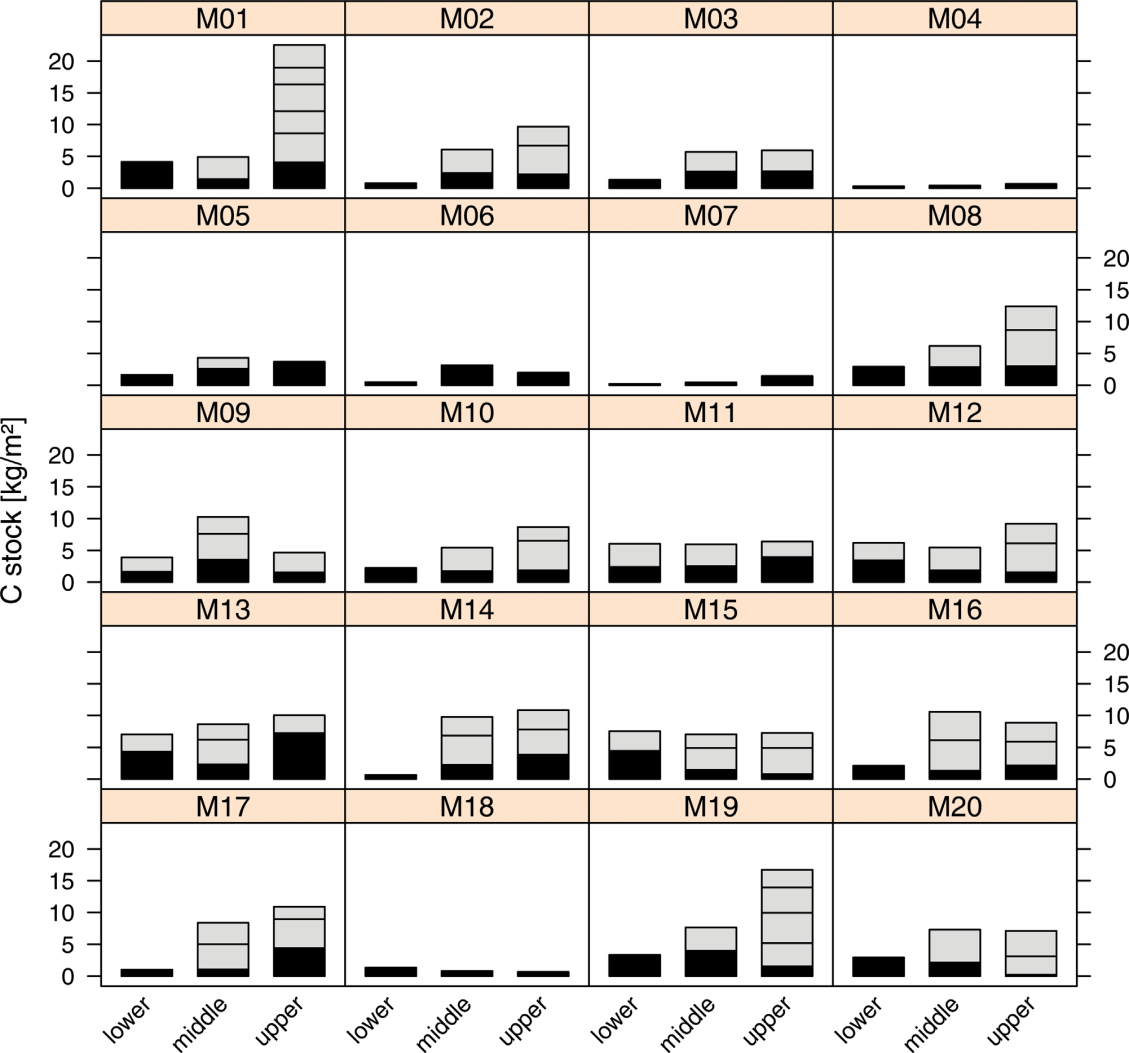
Carbon stocks of the organic layer in each of the sampled profiles at the upper,middle and lower part of the transects M1 to M20. Each part of the stacked bar plots refers to the carbon stock of a layer of 10 cm with exception of the lowermost layer, here indicated by black colour.

Fig 2 gives an overview of the SOC stocks of the organic layer [46] used for this study. Interestingly, the highest stocks are found at the upper slope position of transects M1 and M19, both positioned at rather low altitudes, while transects M4 to M7 which are positioned at high altitudes have very low carbon stocks.

#### 2.2.2 Predictors

Predictors used for model development include parameters derived from a satellite image and a DEM. The Landsat 8 OLI/TIRS image from 2014, February 18^th^, was provided as image courtesy of the U.S. Geological Survey (USGS). The provided image bands were transformed to Top of the Atmosphere Reflectance (TOA) and At-Satellite Brightness Temperature (SatTEMP) using the image’s metadata and information provided by the USGS [47]. The normalised difference vegetation index (NDVI), and the normalised difference moisture index (NDMI) were calculated according to Jackson et al. [48]. The perpendicular vegetation index (PVI) and the transformed soil adjusted vegetation index (TSAVI) are using the soil line concept introduced by Richardson and Wiegand [49]. The soil line concept demonstrates the observed linear relationship between Red and NIR reflectance of bare soil. The parameters β_1_ and β_0_ in Eqs 1 and 2 refer to the slope and intercept of this relation and were automatically determined according to Fox et al. [50] as β_1_ = 0.71 and β_0_ = 0.11. X in Eq 2 is a constant usually assumed to be 0.08 [50] [51] [52].

$$PVI=\frac{1}{\sqrt{\beta_{1}^{2}+1}}\left(NIR-\beta_{1}R-\beta_{0}\right)$$ Eq 1

$$TSAVI=\frac{\beta_{1}(NIR-\beta_{1}R-\beta_{0})}{\beta_{1}NIR+R-\beta_{1}\beta_{0}+X\left(1+\beta_{1}^{2}\right)}$$ Eq 2

All predictors calculated from the mentioned landsat image are shown in Table 1. Landsat bands 1 to 11 refer to the predictor ID 1 to 10, the calculated vegetation indices refer to ID 11 to 14.

**Table 1:** Predictors derived from the landsat image.

ID	Predictor	Input	Calculation
1	Aerosol	band 1	1.2901E-02*x-64.50640
2	Blue	band 2	1.3211E-02*x-66.05534
3	Green	band 3	1.2174E-02*x-60.86943
4	Red	band 4	1.0266E-02*x-51.32853
5	NIR	band 5	6.2821E-03*x-31.41050
6	SWIR1	band 6	1.5623E-03*x-7.81151
7	SWIR2	band 7	5.2658E-04*x-2.63290
8	Panchromatic	band 8	1.1618E-02*x-58.08977
9	TIRS1	band 10	3.3420E-04*x+0.10000
10	TIRS2	band 11	3.3420E-04*x+0.10000
11	NDVI	band 4, band 5	
12	PVI	band 4, band 5	
13	TSAVI	band 4, band 5	
14	NDMI	band 5, band 6	

Geomorphological and hydrological DEM parameters were calculated by R-package RSAGA [53,54]. The DEM was provided by the DFG RU 816 database [55]. Its cell size was adapted to the landsat image’s cell size of 30 m.

The DEM parameters are listed in Table 2. Please be aware of their ID numbers 15 to 236. Predictors 37 to 236 were calculated with different computing window sizes of 3x3 to 41x41 cells corresponding to different degrees of terrain smoothing and search radii of 45 to 615 m.

**Table 2:** Predictors obtained from DEM.

ID	Terrain Parameter	Module Library	Module	Reference	SAGA author/ year
15	Altitude (DEM)	Grid—Spline Interpolation	Multilevel B-Spline Interpolation		Conrad/ 2006
16	Slope		Morphometric Features	[56]	Conrad/ 2013
17	Aspect				
18	Mass Balance Index (MBI)		Mass Balance Index	[57–59]	Conrad/ 2008
19	Slope Height				
20	Valley Depth	Terrain Analysis -	Relative Heights		Böhner &
21	Normalised Height	Morphometry	and Slope Positions		Conrad/ 2008
22	Standardised Height				
23	Wind Effect		Wind Effect		Böhner & Ringeler/2008, Conrad/ 2011
24	Hill Index		Valley and Ridge Detection	[60]	Conrad/ 2013
25	Direct Insolation		Potential Incoming	[61–63]	Conrad/ 2010
26	Diffuse Insolation	Terrain Analysis	Solar Radiation		
27	Visible Sky	- Lighting,	Sky View Factor	[61,62,64]	Conrad/ 2008
28	Sky View Factor	Visibility			
29	Positive Openness		Topographic Openness	[65–67]	Conrad/ 2012
30	Negative Openness				
31	Catchment Area		Catchment Area (Flow Tracing/		
32	Catchment Height	Terrain Analysis -	Kinematic routing algorithm)	[68]	Conrad/ 2001
33	Catchment Slope	Hydrology			
34	SAGA Wetness Index (SWI)	(with pre-processed DEM)	SAGA Wetness Index	[69]	Böhner & Conrad/ 2001
35	Topographic Wetness Index (TWI)		Topographic Wetness Index	[70–72]	Conrad/ 2003
36	LS Factor		LS Factor	[71,73,74]	Conrad/ 2003
37–56	Convergence		Convergence Index (Search Radius)	[75]	Conrad/ 2003
57–76	Terrain Ruggedness Index (TRI)		Terrain Ruggedness Index	[76]	Conrad/ 2010
77–96	Terrain Surface Texture		Terrain Surface Texture	[77]	Conrad/ 2012
97–116	Terrain Surface Convexity		Terrain Surface Convexity	[77]	Conrad/ 2012
117–136	Plan Curvature	Terrain Analysis -			
137–156	Profile Curvature	Morphometry			
157–176	Longitudinal Curvature		Morphometric Features	[78,79]	Conrad/ 2013
177–196	Cross-Sectional Curvature				
197–216	Minimum Curvature				
217–236	Maximum Curvature				

### 2.3 Regression models

Five machine learning algorithms were compared in their performance to predict the organic layer carbon stocks:

1. Random Forest (RF)
2. Artificial neural network (ANN)
3. Multivariate adaptive regression splines (MARS)
4. Boosted regression trees (BRT)
5. Support vector machine (SVM)

#### 2.3.1 Random forest

RF is a recursive partitioning method which grows a number of regression trees [80] and averages the results. In a regression tree the data is subsequently partitioned by the predictor variables into preferably homogeneous subsets regarding the response variable. The mean of each data subset is then used as predicted response value. A partition point or so called node is always defined by that predictor and threshold in its range, which achieves the most homogeneous partition into two subsets (tree branches). At each node all predictors with all possible threshold values are tested.

The RF ensemble model’s stability is obtained through varying the trees’ input dataset and the subset of predictors used to subsequently split the data in each tree node. According to Hastie et al. [24], RF does not require much tuning. The RF model was adapted by R-package “randomForest” [81], growing 1000 trees (ntree) and the models default parameters for the size of the predictor subset (mtry = p/3) and the minimum amount of data in the terminal nodes (nodesize = 5). The size of the data subset selected to grow each tree was set to the size of the training set, sampling was done with replacement.

#### 2.3.2 Artificial neural network

ANNs consist of a number of neurons, the processing units of the algorithm, which are organised in layers. The input–the predictor vectors at each sampling point–has to pass these processing units to relate to the output: the response variable at each sampling point. The neurons of the input layer send data via synapses to the neurons of the first internal layer and these pass it via other synapses to the neurons of the next layer. The synapses store parameters called synaptic weights, which guide the learning process [82]. In general, an ANN is defined by (1) the interconnection pattern between the layers of neurons, (2) the learning rule which updates the weights of these connections, (3) the propagation function that converts a neuron’s weighted input to its output, and (4) the activation function, which determines how the state of the neuron at point of time t+1 is calculated based on its state at point of time t and the new input [83]. Finally, the number of layers and neurons is a crucial decision to be made [36]. Generally one hidden layer is enough to approximate continuous functions, whereas two hidden layers might be necessary in the case of discontinuities [84]. A network with too few neurons cannot differentiate between complex data patterns, whereas too many neurons would lead to overfitting [36]. Data normalisation before ANN training, is essential to prevent larger values from dominating smaller values, which otherwise might lead to premature saturation of neurons [36].

A two layer feed-forward ANN with Bayesian regularisation [85] was trained with function “brnn” of R-package “brnn” [86]. The optimal number of neurons (1 to 20) was selected by five repetitions of an external tenfold cross-validation.

For the all-predictor model the maximum number of possible neurons was reduced to 13 (instead of 20) to combat computation time. With a number of 238 predictors, 13 neurons resulted in the estimation of 3094 parameters (weights and biases) and tuning the number of neurons with a range of 1 to 13 neurons in five times repeated tenfold cross-validation with parallel computing still resulted in a computation time of c. 30 hours. In this particular case the tuning resulted in best performance with only 1 neuron.

#### 2.3.3 Multivariate adaptive regression splines

The model algorithm is constructed by a weighted sum of piecewise linear basis functions of the form (x-t)_+_ and (t-x)_+_. Each of these so called linear splines is defined by a predictor (x) and a node (t), with the latter separating the first linear part of the function from the second linear part [24]. Starting with the intercept (the mean of the response variable), the algorithm repeatedly adds such a spline or a product of two or more splines. To define a new spline, at each step, the algorithm searches all predictors and all values of each predictor. This process of adding splines continues until a threshold value for the change in residual error or the maximum number of terms is reached. Due to its construction of splines with their internal node, MARS resembles recursive partitioning algorithms. However, in contrast to the latter, MARS produces continuous models [87]. Alltogether, the model algorithm also resembles stepwise forward linear regression, but instead of using the predictors themselves as input, it also permits to make use of already included model terms and products thereof [24]. MARS was implemented by R-package “earth” [88], which is based on Friedman’s manuscripts "Multivariate Adaptive Regression Splines" and "Fast MARS" [87,89]. No pruning of the forward pass was applied, but the number of added terms, 1 to 50, was tuned by 5 repetitions of an external tenfold cross-validation. The degree of interaction between predictors was set to 2.

#### 2.3.4 Boosted regression trees

Despite their many benefits, such as intuitive model structure, handling of input data of different measurement level and scale, insusceptibility to irrelevant predictors, insensitiveness to outliers etc., tree models have also disadvantages limiting their performance, particularly in modelling smooth functions, and the dependence of their structure from the input data [90].

Boosting sequentially applies a learning algorithm to repeatedly modified data versions and thereby produces a sequence of simple models, whose predictions are finally combined to make the overall prediction [24]. In BRT, at first a regression tree is constructed by assigning all training observations the same weight. Then at each iteration step a new regression tree is trained by giving those observations which impaired model performance in the previous step a higher weight [20]. In this way a sequence of models is build. The resulting BRT model is similar to an additive regression model with the subsequently fitted regression trees as individual terms [90].

BRT was implemented with R-package “gbm” [91], which is based on Freund and Schapire’s AdaBoost [92], using a maximum number of 500 iterations. The optimal number of iterations was then selected by five repetitions of a tenfold cross-validation. Shrinkage, a crucial parameter refering to the learning rate was set to 0.001. The data subsampling rate for each of the trees which incorporates a random effect into the BRT algorithm and provides, therefore, the chance to improve BRT model performance [93], was set to 0.90. From the two parameters determining the size of each of the regression trees, interaction depth was set to 2 and the minimum number of samples in each of the final subsets was set to 5.

#### 2.3.5 Support vector machine

SVMs were originally developed as an algorithm for classification, which seperates the classes like other machine learning algorithms by a hyperplane. However, in contrast to other algorithms it searches for that hyperplane which leaves the largest possible margin between classes free of data, leading to a better generalisation probability [94]. Each object to be classified is represented by a vector; the distance of those vectors closest to the hyperplane is maximised. Consequently, only the vectors close to the hyperplane are important for defining it, and are, therefore, called support vectors [94]. Points on the correct side and far away from it are ignored in the process of finding the best hyperplane.

In support vector regression (SVR) there is an analogy in that these “low-error-points” are those with small residuals [24]. The margin of a regression line shall contain all points with errors smaller than ε, i.e. no importance is given to these errors as long as they are smaller than ε [95]. SVR searches for a function which fulfils this ε-criterion and is as flat as possible [96]. Points outside the margin are allowed while a penalty weight C is introduced. It determines the trade-off between allowing points outside ε and the flatness of the regression function [96]. This is particularly important to decrease the impact of outliers. Non-linear regression is typically achieved by applying the so-called Kernel trick. Here, the training data is first transformed into a higher-dimensional feature space by applying a non-linear kernel function. Then a linear model is adapted to this new feature space [97]. Finally, the linear regression in the new feature space is equivalent to a non-linear regression in the original predictor space.

Data normalisation before training a SVM is very important to avoid attributes of higher ranges dominating those with smaller ranges and to avoid problems when calculating the kernel function [37]. The support vector regression model parameters C and ε and the kernel parameters must be tuned to obtain sensible results [37,38]. SVR with a gaussian radial basis function kernel $f\left(x\right)=e^{\left(-\gamma {|x-v_{i}|}^{2}\right)}$ was applied by R-package “e1071” [98]. The width parameter *γ*, the inverse of the radius of influence of the selected support vectors *v*_*i*_, determines the amount of generalisation of the model. According to Cherkassky and Ma [38] $\varepsilon =3\sigma \sqrt{\text{ln}\left(n\right)/n}$ gives good performance. C and the radial kernel parameter *γ* were adapted by five repetitions of a tenfold cross-validation. For C a range of 0.1 till 1.0 with a step size of 0.1 was chosen, following Mattera and Haykin [99] and Cherkassky and Ma [38]. For *γ* a range of 0.2 till 0.5 with a step size of 0.05 was chosen [38].

### 2.4 Model validation and comparison

Five- or tenfold cross-validation are usually recommended to test model performance (e.g. [100,101]). The decision on how the subsets are formed remains important as the formed subsets may have an impact on the cross-validated error [100]. Subsets were formed by random selection. However, to account for the impact of subset membership, tenfold cross-validation was repeated five times with different subsets. Parallel computing of the five repetitions was employed with r-packages “doParallel” and “foreach” [102,103] to combat computation time. Predictors as well as response variable where normalised prior to model training for all five model algorithms to allow for comparison regarding their predictive performance; the scaling default setting, which exists in some of the models (ANN, MARS and SVM), was turned off.

### 2.5 Predictor selection

Wrapper methods developed for multiple regression models include forward selection, backward elimination, stepwise selection and best subsets. The forward selection procedure usually starts with the predictor most highly correlated with the response variable and tests if the resulting model is significant. It then repeatedly adds predictors at each step, testing for model improvement due to the significance of the calculated F-statistic [19]. We applied a slightly different approach to forward selection: At first all individual predictor models of the five machine learning algorithms were compared in performance by their repeated cross-validated RMSE resulting in a predictor ranking. The single predictor included in the best individual predictor model was then used to start the forward selection procedure. At the end of step 1 it was tested whether the various algorithms’ parameter ranges to select the optimal parameter via cross-validation were reasonable or needed to be adapted.

**Fig 3:**
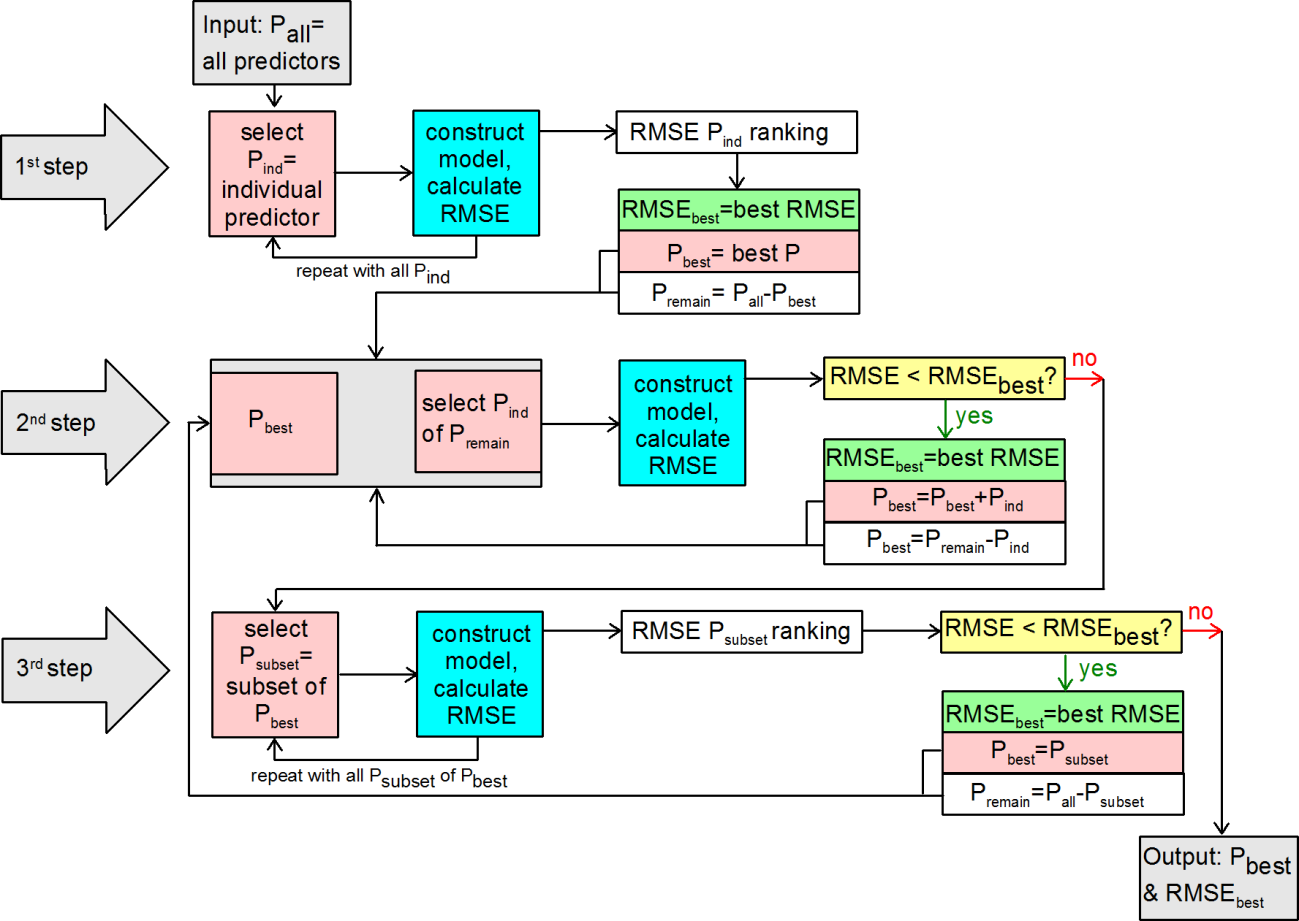
Forward selection procedure for predictor subset selection.

The model including the ten best predictors of the predictor ranking obtained after step 1 (Fig 3) was compared to two forward selection procedures: Simple forward selection starting with the best predictor (as selected by the best individual predictor model) and a three step forward selection procedure. Fig 3 includes all three procedures:

1. Predictor ranking at the end of step 1 (Fig 3) based on all individual predictor models and their repeatedly cross-validated RMSE. Build model of the 10 best predictors as selected by the ten best individual predictor models: 10bestPR.
2. Simple forward selection (sFS) includes steps 1 and 2 (Fig 3).
3. Three-step forward selection (3stepFS) included all three steps and repeats steps 2 and 3 until no further improvement in repeated cross-validated RMSE is achieved.

## 3 Results and Discussion

### 3.1 Selected predictors

Table 3 gives an overview of the selected predictors for each machine learning algorithm (please compare Tables 1 and 2 for predictor IDs). The selected predictors are ordered according to their individual importance (predictor ranking) or order of inclusion during the selection procedures. The best individual predictor is predictor 22 “Standardised Height” in all five model algorithms. As the sFS procedure starts with the best individual predictor, this first predictor is the same as in the model ranking (10bestPR). In two cases this best predictor is removed while applying 3stepFS, the two tree algorithms: RF and BRT. Interestingly, most of the ten single most important predictors (10bestPR) are not included in sFS: only one predictor for RF and MARS, two for ANN and BRT, and three for SVM (indicated by surrounding boxes and bold style in Table 3). For MARS and SVM the 3stepFS procedure did not lead to any improvement compared to sFS, which is why the selected predictors are the same. In the three other model algorithms 3stepFS resulted in an improvement in predictive performance and a reduced number of predictors.

**Table 3:** Selected predictors in order of importance.

Model			RF			ANN			MARS			BRT			SVM	
Selection Proc.		1	2	3	1	2	3	1	2	3	1	2	3	1	2	3
Predictors	1	**22**	**22**	236	**22**	**22**	22	**22**	**22**	22	**22**	**22**	26	**22**	**22**	22
	2	174	236	15	18	21	217	30	97	97	**26**	**26**	198	37	**26**	26
	3	37	15	198	97	148	26	18	36	36	15	198	148	18	148	148
	4	119	198	169	37	**217**	20	153	145	145	5	148	47	**26**	217	217
	5	97	20	26	38	26	225	20	156	156	37	47	118	**15**	**15**	15
	6	153	169	103	33	20	65	197			175	118	219	12	119	119
	7	5	26		235	225	10	33			119	219	73	174	121	121
	8	175	103		236	65	167	198			174	73	178	198		
	9	178	149		39	10	124	23			30	178	171	38		
	10	155			**217**	40		37			155	171	150	217		
	11					138						150				
	12											24				
	13											14				

Predictor selection: 1 = 10bestPR, 2 = sFS, 3 = 3stepFS, predictor IDs are listed in order of importance from 1 to 13

Table 4 includes only those predictors of Tables 1 and 2 which have been selected at least once. To summarise, similar predictors were grouped. This shows that there are many predictors which describe terrain curvature in one way or another, such as Hill Index, Convergence, TS Convexity and the various types of curvature. Filled table cells indicate whether a certain predictor was selected by the predictor selection procedure in the particular machine learning algorithm. The numbers in some of the cells refer to the search radii of the selected predictors. The table shows that sometimes a particular predictor was included in different radii in the forward selection process compared to the 10best predictor ranking. All together convergence and curvature parameters were always among the selected predictors. Another often chosen predictor was “Diffuse Insolation”. The common best predictor for all machine learning algorithms was “Standardised Height”, a predictor referring to the slope position.

**Table 4:** Overview of selected predictors.

Algorithm		RF			ANN			MARS			BRT			SVM		
Predictor selection		1	2	3	1	2	3	1	2	3	1	2	3	1	2	3
group	predictor															
	NIR	X									X					
satellite	TIRS2					X	X									
data	PVI													X		
	NDMI											X				
altitude	altitude		X	X							X			X	X	X
MBI	MBI				X			X						X		
slope	Valley Depth		X			X	X	X								
position	Norm. Height															
	Stand. Height	X	X		X	X	X	X	X	X	X	X		X	X	X
lighting,	Diff. Insolation		X	X		X	X				X	X	X	X	X	X
visability,	Neg. Openness							X			X					
wind	Wind Effect															
hydrology	catchm. Slope				X			X								
	LS factor								X	X						
TRI						285	285					525	525			
	hill index											X				
		45			45	135		45			45	345	345	45		
	Convergence				75									75		
					105											
	TS Convexity	45	225	225	45				45	45						
	Plan Curv.	105					255				105	75	75		105	105
conver-															165	165
gence/	Prof. Curv.	525	405			75		17	285	285	585	375	375		375	375
curvature		585				375			615	615		435	435			
	Long. Curv.	555	405	405			345				555	465	465	555		
		585									585					
	Cross.-Curv.	75										75	75			
	Min Curv.		75	75				1				75	75	75		
								2								
			615	615	45	45	45					105	105	45	45	45
	Max Curv.				585	285	285									
					615											

predictor selection: 1 = 10bestPR, 2 = sFS, 3 = 3stepFS

filled cells indicate selected predictors, numbers in cells indicate the applied GIS search radius in meters

Altitude was only important for RF, BRT and SVM, but not for ANN and MARS. Satellite data was important for all algorithms except MARS. The predictor “diffuse insolation” was among the final predictor set (3stepFS selection) in four of the five algorithms. Interestingly, MBI was among the 10bestPR in ANN, MARS and SVM but never selected in any of the two selection procedures in any of the five algorithms. From the hydrological parameters only catchment slope and LS Factor were of any relevance for ANN and MARS.

### 3.2 Model performance

**Fig 4:**
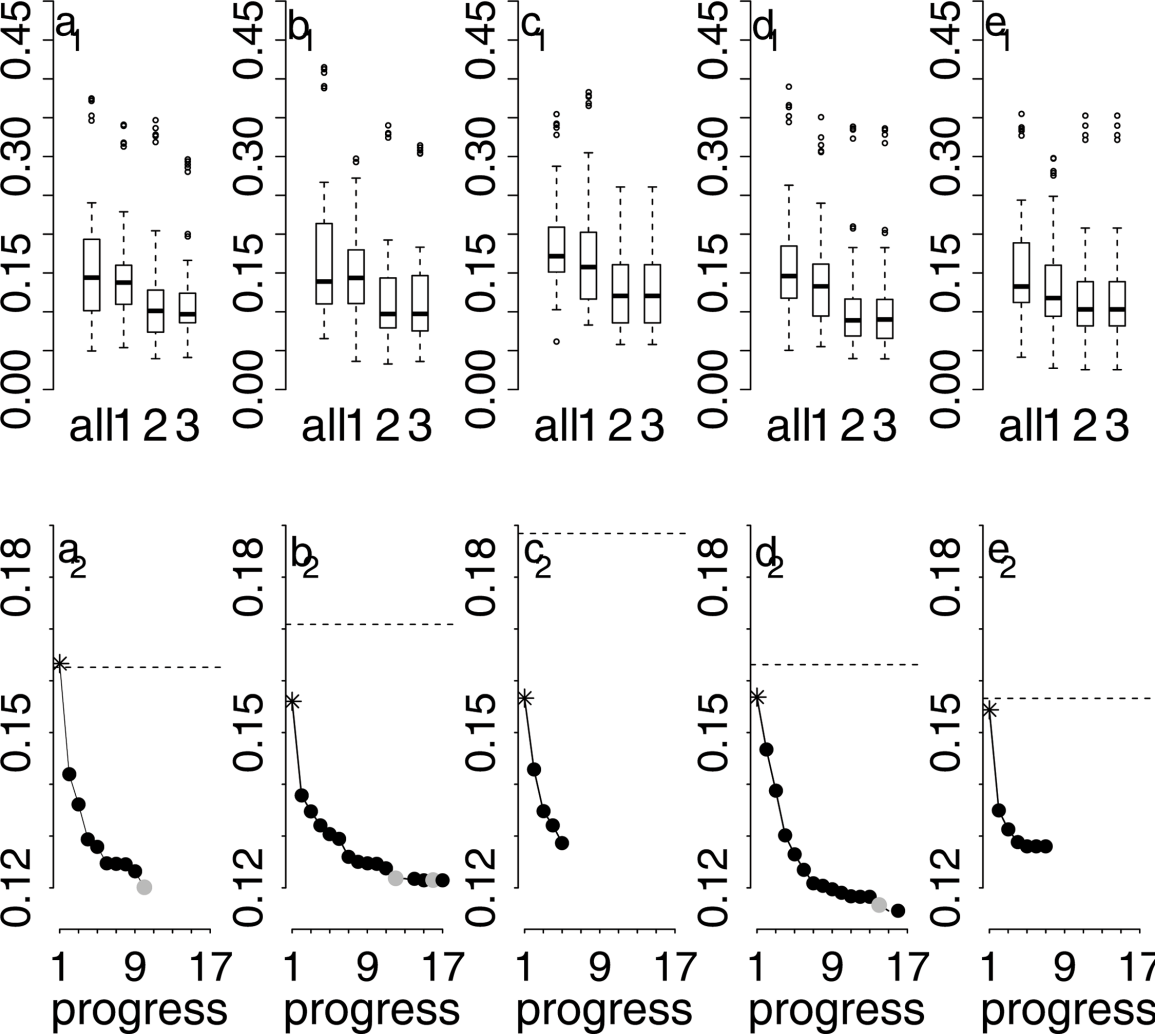
RMSE boxplots of repeated cross-validation (a_1_-e_1_) and development of the mean RMSE of rpeated cross-validation during the predictor selection process (a_2_-e_2_). a) RF, b) ANN, c) MARS, d) BRT, e) SVM. In a_1_-e_1_: “all” refers to the all-predictor model, “1”refers to the 10bestPR model, “2” to the sFS model, and “3” to the 3stepFS model. In a_2_-e_2_: The star refers to the mean RMSE of the best individual predictor model of step 1, black points refer to added predictors and the resulting mean RMSE during step 2, and grey points refer to the mean RMSE after step 3. The dashed line represents the mean RMSE of the all-predictor model.

Fig 4A_1_–4E_1_ compare the results of the three selection procedures (1, 2, 3) for the five machine learning algorithms to the all-predictor model (all) results. The figures show the RMSE boxplots of the repeated cross-validation. Please be aware that the RMSEs refer to the normalised C stock data.

The 10bestPR predictor selection improved predictive performance compared to the all-predictor model in all but the ANN algorithm. Applying the sFS procedure improved it even further and in all five algorithms. As mentioned before, in two algorithms, MARS and SVM the subset selection (step 3 in Fig 3) did not improve model results. Therefore, the RMSE boxplots 2 and 3 of Fig 4C_1_ and 4E_1_ are exactly the same. But even for ANN and BRT there was only a very slight improvement (Fig 4B_1_ and 4D_1_); the median and the interquartile range of the RMSE boxplots are very similar. This improvement is somewhat more pronounced for RF, particularly while comparing the interquartile ranges of RMSE boxplot 2 and 3 (Fig 4A_1_).

Fig 4A_2_–4E_2_ show the development of the repeatedly cross-validated mean RMSE during the three-step selection procedure. Here the dashed line represents the repeatedly cross-validated mean RMSE of the all-predictor model. The star represents the same error measure regarding the best individual predictor model (step 1, Fig 3). The hereby selected first predictor is then entering the simple forward selection procedure (step 2, Fig 3). The first black point refers to the error measure of the model with the first additional predictor (step 2, Fig 3). The number of this first sequence of black points corresponds to the number of predictors added in step 2 before no further improvement can be achieved in sFS. Then the grey point represents the error measure of the best subset selected in step 3 (Fig 3). Black points after the grey point indicate that another sequence of simple forward selection (step 2) is applied and so on. For RF (Fig 4A_2_) it was enough to run the three steps once, BRT (Fig 4D_2_) repeated step 2 a second time, ANN needed to repeat step 2 a third time (Fig 4B_2_). Finally, applying the 3stepFS to BRT resulted in the best predictive performance with a mean RMSE of 0.116, corresponding to a ten-predictor-model (please compare Table 3). However, RF and ANN performed only slightly worse with a mean RMSE of 0.120 and 0.121, respectively. Particularly, the RF model has the benefit of being much less complex with a number of only six predictors (Table 3). Interestingly, the one-predictor model improved model performance compared to the all-predictor model in all but one machine learning algorithm (RF). For MARS this improvement was comparatively high. In all five considered machine learning algorithms sFS resulted in an improvement in the predictive performance. This improvement was particularly pronounced for the three recursive partitioning algorithms: RF, MARS and BRT. Usually, recursive partitioning methods are said to be rather resistant to non-informative predictors [23]. They choose the best predictor at each split and should, therefore, theoretically be resistent to irrelevant predictors. However, adding irrelevant predictors to a classification tree algorithm also resulted in a deterioration by 5 to 10% on datasets tested by Witten and Frank [18]. Witten and Frank [18] also explain why even relevant attributes can cause harm in recursive partitioning algorithms due to the possibly highly unbalanced subdivision into two subsets. According to Kuhn and Johnson [23], ANN and SVM are even affected to a much larger extent by irrelevant predictors, which could not be confirmed by our results.

With a high number of non-informative predictors, sFS will always result in an improvement even after few iterations steps. And a number of non-informative predictors is likely to be tested in situations were the predictor-response correlation is weak. During model parameter tuning we further realised that the level of improvement is controlled by the algorithms’ fine tuning. BRT for example resulted in the overall worst model while only 500 iterations were computed, but in the best with the final 10,000 iterations. At the end of step 1 it was tested whether the various algorithms’ parameter ranges to select the optimal parameter via cross-validation were reasonable or needed to be adapted. Accordingly, the upper and lower limit of the SVM gamma and C parameter ranges needed to be adapted with the new range from 10^−3^ to 10^4^. A higher range of neurons might have resulted into a slightly better RMSE. However, we refrained from doing so since it tremendously increased computation time of the already most time consuming machine learning algorithm. To allow for practicability, the amount of parameter tuning was set to reduce the very time-consuming forward selection (step 2) to a maximum of 12 hours (with parallel computing). This means we do not know if particularly ANN and SVM could be improved even further by testing smaller steps in the C and gamma ranges of SVM and a higher number of neurons in ANN.

### 3.3 Predicted carbon stocks

**Fig 5:**
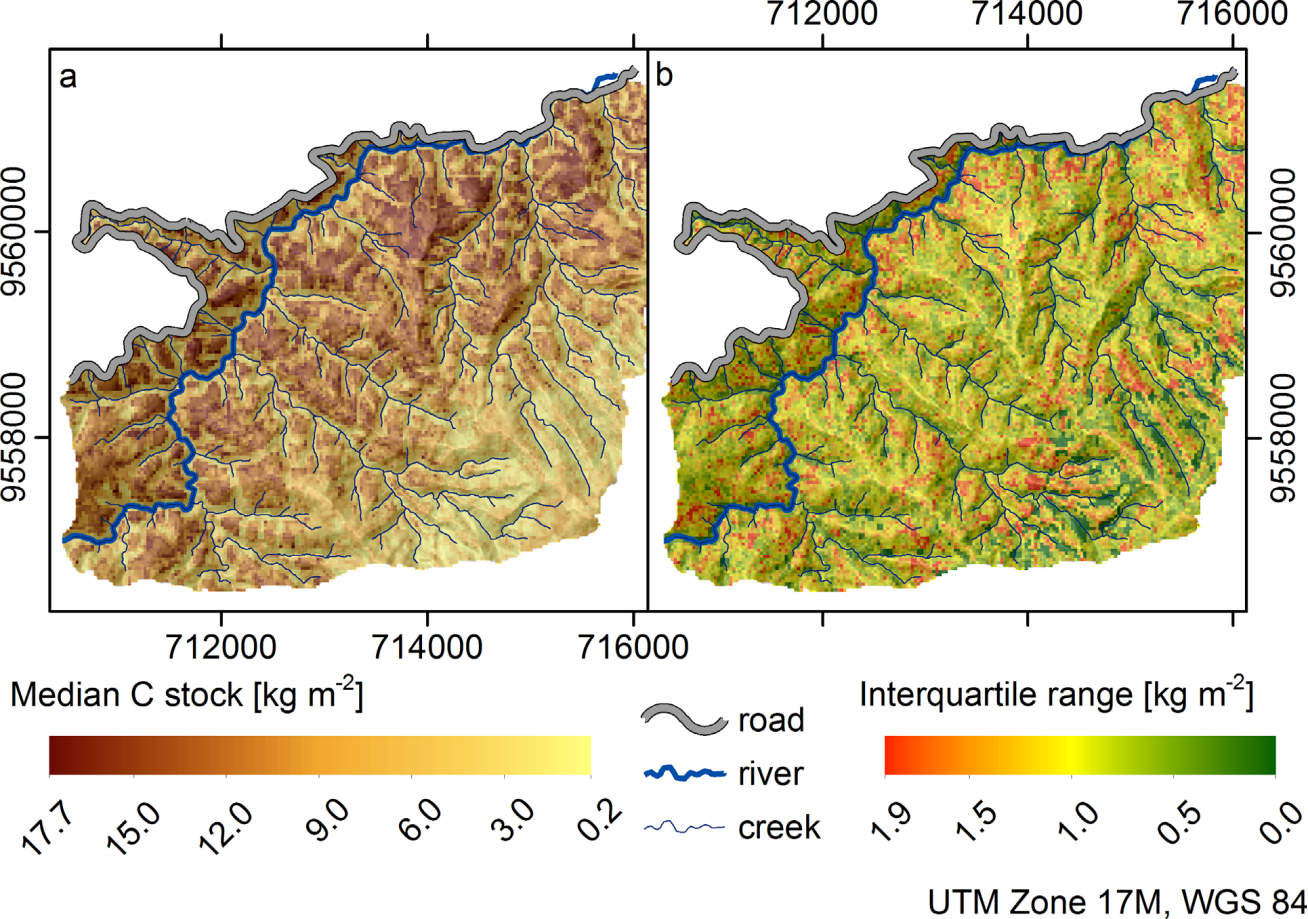
SOC stock prediction with best model BRT 3stepFS. a) Median prediction value, b) Interquartile range (overlaid hillshading with light source from north).

Fig 5A shows the median carbon stocks of the soil organic layer under tropical mountain forest and páramo vegetation as predicted by the overall best model: BRT with 3stepFS. The area north of the interprovincial road (Fig 1) was not predicted due to lack of data for this area of high disturbance by fire and non-natural vegetation (pasture). The majority of studies assessing SOC in tropical forests have been conducted in lowland forests, while the SOC stocks of tropical mountain forests are less well known [104]. Moser et al. [104] investigated SOC in two plots of 20 x 20 m within our research area (at 1890 and 2380 m a.s.l.) and report 3.7 to 4.8 Kg m^-2^ SOC in the organic layer which lie within the lower range of our findings. The corresponding interquartile range of our predictions (Fig 5B) is below 1 kg m^-^² for 90% of the area indicating a low prediction uncertainty due to the underlying position specific density function in areas of high SOC stocks. Accordingly, the relative prediction uncertainty in areas of low SOC is higher; rainfall induced landslides, frequently occurring within the area, certainly reduce predictability. The apparently lower organic carbon stocks above an altitude of about 2500 m a.s.l. coincide with a change from mountain forest to shrub and páramo vegetation and the corresponding shallower organic layers (1 to 15 cm) observed during sampling: Transect M4 to M7 (Fig 1). In general, median organic layer thickness varies between c. 20 to 70 cm throughout the area, with organic layer thickness being highest on mid-slope positions [10].

According to Roman et al. [105], the high SOC stocks of tropical montane cloud forests cannot only be explained by low soil organic matter turnover due to slow rates of litter decomposition, soil acidity and reduced rates of nutrient cycling. They conclude that the organic soil layer of these ecosystems is highly variable. Furthermore, soil water logging and the altitudinal gradient alone cannot explain the complex spatial pattern [10]. SOC stocks in tropical forest soils of Papua New Guinea are reported to vary between 4.8 and 19.4 kg m^-2^ (litter and top 100 cm) with an increase of 5.1 kg m^-2^ per 1000 m increase in altitude, while SOC in litter stayed the same [106]. Don et al. [4] report SOC stocks between 7.3 and 10.5 kg m^-2^ in tropical primary forests. They analysed 385 studies on SOC from tropical countries but had to exclude data on organic layer SOC due to dataset scarcity.

The selected predictors which influence the spatial variation include diffuse insolation, topographic ruggedness index, convergence and all considered curvatures (some in different search radii). Diffuse insolation and topographic ruggedness index are related to site exposure, a factor termed important by Roman et al. [105] when studying the global and local variations in tropical montane cloud forest soils and particularly the accumulation of soil organic matter. The low search radii for plan curvature, cross-sectional curvature, minimum and maximum curvature (75 and 105 m) indicate the importance of local curvatures, while the rather high search radii for the profile and longitudinal curvature (375 to 465 m) indicate the importance of the larger landscape topography. The influence of the diffuse insolation and the influence of the ridge and valley topography on the spatial carbon stock pattern are clearly visible in Fig 5. Carbon stocks are lower in areas of high diffuse insolation (Summit area in the South-East) and in the concave valley structures. Similarly, Wilcke et al. [107], who studied transects in the lower part of the here investigated area, also report higher humus concentrations on the ridge-tops as compared to the valley structures and explain this with stronger acidification and nutrient leaching on the exposed sites. Gessler et al. [27] report flow accumulation being a good predictor for SOC. According to them, ignoring the effect of topography presents a serious weakness in approaches to regional and global carbon modelling, as water distribution between convex and concave landscape positions may account for differences in SOC similar to dramatically different climatic zones. According to Grimm et al. [25], topographical parameters approximate water and solute transport, relate to solar insolation and determine the micro-climate. In that effect, they have the potential to explain large parts of SOC variation.

Predictors obtained from satellite images were not among the selected predictors for the best model: According to Waring and Running [108] multi-spectral reflectance data obtained from satellite images can provide valuable information on forest structure and productivity. However, in forested areas, remote sensing methods are of limited utility for soil studies due to the variable effects of vegetation [12].

## 4 Conclusions

It was shown, that particularly in the complex soil-landscape setting of tropical mountain areas with possibly low predictor-response correlations, the applied methodology for spatial prediction is of high importance to improve the expected poor model results. Considering different spatial settings/scales for the predictors as well as applying predictor selection and model tuning are important to improve predictive performance.

Concerning predictor selection, choosing predictors individually was not the best option. The forward selection resulted in better predictions than the 10bestPR, emphasizing the importance of predictor interaction. Among the forward selection procedures, the tested 3stepFS algorithm did not result into much improvement compared to the sFS. However, the amount of improvement in predictive performance due to predictor selection depended on model tuning. Finally, even a single- or two-predictor model was better than the all-predictor model independent of the applied machine learning algorithm. Predictor selection is, therefore, also important for recursive partitioning algorithms which are often reportet to be immune to non-informative predictors. To save computation time in the case of many (>100) predictors we recommend to rather apply forward instead of backward selection. Choosing the initial best starting predictor by running the model and not due to correlation seems favourable to make the approach more consistent.

Predictors obtained from satellite images were not among the selected predictors for the best overall model. We assume that satellite images are most probably of higher importance in predicting SOC in areas with contrasting land use types. The highest C stocks appear at the ridges and mid-slope positions at altitudes below 2500 m a.s.l.. Various curvatures in different search radii and the diffuse insolation are best suited to describe this pattern. The interquartile range show slightly bigger uncertainties in the transition zone from montane forest to paramo vegetation at 2500 m a.s.l.. One might argue that other predictors would lead to even better results. However, in most tropical mountain regions it will be difficult to obtain additional predictors apart from the here presented.
